# Biomechanical comparison of the femoral neck system versus InterTan nail and three cannulated screws for unstable Pauwels type III femoral neck fracture

**DOI:** 10.1186/s12938-022-01006-6

**Published:** 2022-06-10

**Authors:** Zheng Wang, Yong Yang, Gangning Feng, Haohui Guo, Zhirong Chen, Yaogeng Chen, Qunhua Jin

**Affiliations:** 1 Department of Orthopaedic, General Hospital of Ningxia Medical University, Yinchuan, 750004 Ningxia China; 2 Ningxia Medical University, Yinchuan, 750004 Ningxia China

**Keywords:** Pauwels type III femoral neck fracture, Biomechanical properties, Internal fixation, Femoral neck system, Artificial composite femur bone

## Abstract

**Background:**

There are a variety of internal fixation methods for unstable femoral neck fractures (FNFs), but the best method is still unclear. Femoral neck system (FNS) is a dynamic angular stabilization system with cross screws, and is a new internal fixation implant designed for minimally invasive fixation of FNFs. In this study, we conducted a biomechanical comparison of FNS, InterTan nail and three cannulated screws for the treatment of Pauwels III FNFs and investigate the biomechanical properties of FNS.

**Methods:**

A total of 18 left artificial femurs were selected and randomly divide into Group A (fixation with FNS), Group B (fixation with InterTan nail) and Group C (fixation with three cannulated screws), with 6 specimens in each group. After creating Pauwels type III FNF models, the specimens in each were tested with non-destructive quasi-static tests, including torsion, A-P bending and axial compression tests. The average slope of the linear load–deformation curve obtained from quasi-static tests defines the initial torsional stiffness, A-P bending stiffness, and axial compression stiffness. After cyclic loading test was applied, the overall deformation of models and local deformation of implant holes in each group were assessed. The overall deformation was estimated as the displacement recorded by the software of the mechanical testing apparatus. Local deformation was defined as interfragmental displacement. Data were analyzed by one-way analysis of variance (ANOVA) followed by Bonferroni post hoc test using the SPSS software (version 24.0, IBM, New York, NY, USA). Correlation analysis was performed using Pearson’s correlation analysis.

**Results:**

Group B exhibited significantly higher axial stiffness and A-P bending stiffness than the other two groups (*P* < 0.01), while Group A had significantly higher axial stiffness and A-P bending stiffness than Group C (*P* < 0.01). Groups A and B exhibited significantly higher torsional stiffness than Group C (*P* < 0.01), no statistical significance was observed between Groups A and B (*P* > 0.05). Group B exhibited significantly lower overall and local deformations than the other two groups (*P* < 0.01), while Group A had significantly lower overall and local deformations than Group C (*P* < 0.01). Correlation analysis revealed positive correlation between axial stiffness and A-P bending stiffness (*r* = 0.925, *P* < 0.01), torsional stiffness (*r* = 0.727, *P* < 0.01), between torsional stiffness and A-P bending stiffness; negative correlation between overall, local deformations and axial stiffness (*r* = − 0.889, − 0.901, respectively, both *P* < 0.01), and positive correlation between the two deformations (*r* = − 0.978, *P* < 0.01).

**Conclusion:**

For fixation of unstable FNFs, InterTan nail showed the highest axial stiffness and A-P bending stiffness, followed by FNS, and then three cannulated screws. Torsional stiffness of FNS was comparable to that of the InterTan nail. FNS, as a novel minimally invasive implant, can create good mechanical environment for the healing of unstable FNFs. Clinical studies are needed to confirm the potential advantages of FNS observed in this biomechanical study.

## Introduction

The incidence of femoral neck fractures (FNFs) accounts for 50% of all hip fractures, and is considered a major public health problem with high socioeconomic burden [1]. Although internal fixation is preferred over hip arthroplasty for treatment of FNFs in non-elderly patients, but there is no consensus regarding the choice of optimal fixation methods. A variety of internal fixation methods has been used to treat FNFs, but it has been reported that the incidence of related complications during the healing process following surgical treatment ranges from 15 to 40% [2, 3]. Nonunion and femoral head necrosis are two major complications after internal fixation. Fracture healing can be classified as either primary or secondary bone healing [4]. FNFs can only be healed by primary bone healing because the femoral neck lacks a periosteal covering [5]. Primary bone healing requires anatomical reduction and a stable fixation, maintaining reduction during fracture healing process is very important [6]. The varus tilting, rotational deformities or retroversion of the head–neck fragment caused by insufficient stability of internal fixation can ultimately lead to nonunion fracture [7–9].

During the treatment of FNFs, with the establishment of anatomical reduction, the maintenance of fracture stability largely relies on internal fixation. The ability of the internal fixation device to provide good stability becomes a key factor in maintaining fracture reduction and stabilization. Pauwels type III FNFs are considered more unstable due to the high shear stress of these fractures, so it is required that implants used for fixation of Pauwels type III FNFs should provide optimal mechanical resistance [10, 11]. Internal fixation with traditional multiple cannulated screws or sliding hip screws are the methods preferred by most surgeons [12]. Three parallel cannulated screws can be used to treat unstable and displaced subcapital or transcervical FNFs. Although fixation with three parallel hollow screws confer less stability than fixation with sliding hip screws, but it is still preferred by many surgeons because fixation with 3 parallel hollow screws do not require a large incision and more extensive soft tissue dissection, and it is simple, minimally invasive and inexpensive [13]. The femoral neck system (FNS) is a new implant system designed for minimally invasive fixation of FNFs in recent years. FNS is instrumented with a dynamic anti-rotation screw to ensure sliding compression and provide rotational stability, an angle plate to provide angular stability [14]. FNS can be used as a treatment option for Pauwels type III FNFs because it is superior to cannulated screws in terms of resistance against varus deformity, and is comparable to sliding hip screws in terms of sustainability of the restored neck length [14]. InterTan nail was an intramedullary device with an intramedullary nail and two integrated cephalocervical screws. This device differs considerably from existing implants in that it allows for immediate intraoperative compression of the principal fracture fragments through linear compression combined with rotational stability secondary to its unique geometry and mechanism of action [15]. Rupprecht et al. [16] confirmed that compared with cannulated screws and dynamic hip screw (DHS), intramedullary fixation device, InterTan nail was more powerful for fixation of Pauwels type III FNFs.

However, the effect of FNS for fixation of Pauwels type III FNFs in terms of axial stiffness, anteroposterior (A-P) bending and torsional stiffness has not yet been compared with cannulated screws and InterTan nail. Therefore, the purpose of this study was to compare the difference in biomechanical properties between FNS, cannulated screw and InterTan nail for fixation of Pauwels III FNFs using non-destructive loading test. We hypothesize that FNS may improve fracture stability.

## Results

### Biomechanical test results

As shown in Table 1, there were significant differences in the axial stiffness and A-P bending stiffness between three groups (both *P* < 0.01). Group B exhibited the highest axial stiffness and A-P bending stiffness, followed by group A, and then Group C.

**Table 1:** Biomechanical test results of three fixation methods

Group	Axial stiffness (N/mm)	A-P bending stiffness (N/mm)	Torsional stiffness (Nm/°)	Overall deformation (mm)	Local deformation of implant holes (mm)
Group A	423.71 ± 18.69	227.46 ± 26.07	0.32 ± 0.01	2.45 ± 0.25	1.2 ± 0.1
Group B	485.63 ± 13.27^a^	410.08 ± 50.37^a^	0.31 ± 0.03	2.02 ± 0.10^a^	0.81 ± 0.08^a^
Group C	375.75 ± 14.32^a,b^	145.2 ± 14.83^a,b^	0.22 ± 0.02^a,b^	3.39 ± 0.23^a,b^	2.18 ± 0.07^a,b^
*F*	74.781	96.280	45.222	70.550	435.023
*P*	< 0.001	< 0.001	< 0.001	< 0.001	< 0.001

^a^*P* < 0.01, vs. Group A; ^b^*P* < 0.01, vs. Group B

Values are presented as mean ± standard deviation

*F* and *P* values are obtained by one-way analysis of variance

Different letters (a, b) indicate significant differences in the same column at a *P* < 0.01 by Bonferroni post hoc test

There were no statistical significance in the torsional stiffness between group A and B. Torsional stiffness was comparable for FNS and InterTan nail, whereas the lowest torsional stiffness was observed in Group C.

**Fig. 1:**
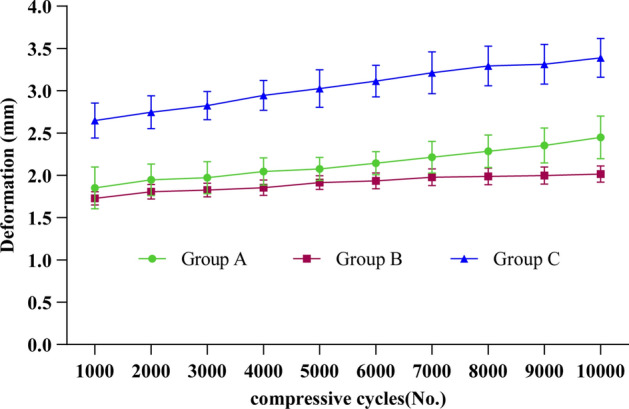
Load–deformation curves. During the dynamic cyclic loading test, InterTan nail (Group B) provided increased stability compared with FNS (Group A) and three cannulated screws (Group C). Three cannulated screws provided less stability than FNS and InterTan nail

**Fig. 2:**
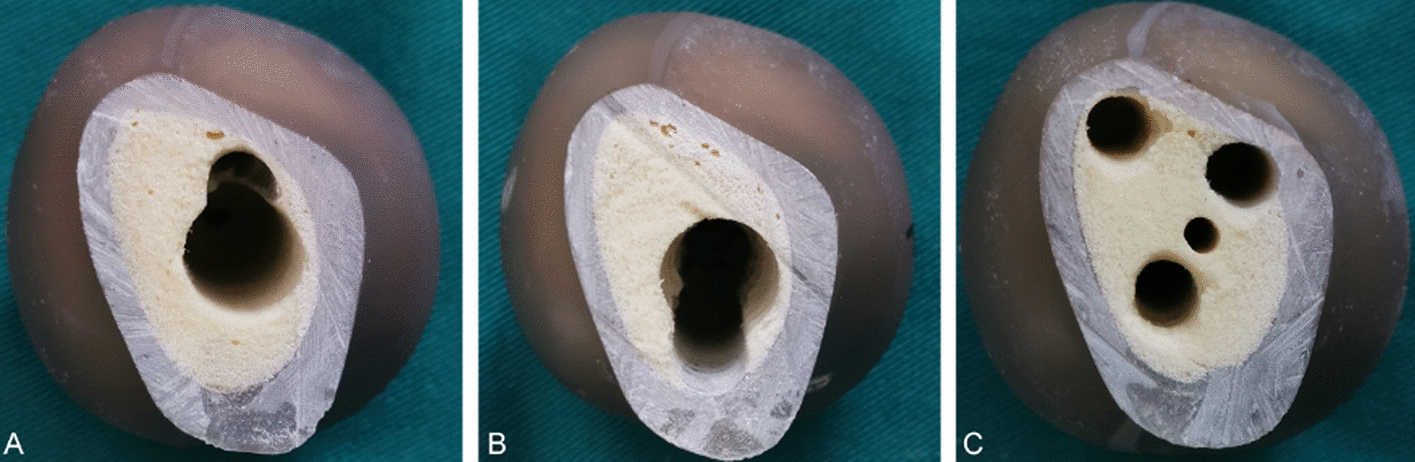
Deformation of holes in the distal-to-proximal direction. **A** FNS hole, **B** InterTan nail hole; **C** cannulated screw holes. No significant deformation of the InterTan nail hole was seen, indicating that InterTan nail had highest stability. Slight extrusion was visible at the distal and proximal direction of the FNS holes. All three cannulated screw holes showed deformation to varying degrees, with the most pronounced deformation below the calcar femorale

Group B exhibited better biomechanical stability than Group A and C in the cyclic loading test (Fig. 1). There were significant differences in the average overall deformation and deformation of holes in the distal–proximal direction between three groups (both *P* < 0.01, Table 1, Fig. 2). Group C exhibited the highest overall and local deformations, followed by Group A, and then Group B. All models had withstood loading without fracture and fixation failure.

### Correlation analysis results

Correlation analysis revealed positive correlation between axial stiffness and A-P bending stiffness (*P* < 0.01), torsional stiffness (*P* < 0.01), as well as between torsional stiffness and A-P bending stiffness (*P* < 0.01, Table 2).

**Table 2:** Correlation between axial stiffness, A-P bending stiffness and torsional stiffness

Indicators	Pearson correlation coefficient
Axial stiffness VS A-P bending stiffness	0.925^**^
Axial stiffness VS torsional stiffness	0.727^**^
Torsional stiffness VS A-P bending stiffness	0.616^**^

^**^P < 0.01

Negative correlation was found between overall, local deformations and axial stiffness (both *P* < 0.01), while positive correlation was found between the two deformations (*P* < 0.01, Table 3).

**Table 3:** Correlation between axial stiffness and overall, local deformations

Indicators	Pearson correlation coefficient
Axial stiffness VS overall deformation	− 0.889^**^
Axial stiffness VS local deformation	− 0.901^**^
Overall deformation VS local deformation	0.978^**^

^**^P < 0.01

### Discussion

To the best of our knowledge, this study is the first to compare the biomechanical properties of FNS with traditional cannulated screws and InterTan nail for treating unstable FNFs. We conducted biomechanical comparison of FNS, traditional cannulated screws and intramedullary fixation device (InterTan nail) for fixation of Pauwels type III FNFs. Our results showed that InterTan nail exhibited highest axial stiffness and AP bending stiffness, followed by FNS, and then 3 cannulated screws. This may be due to that during axial compression and AP bending, fixation with InterTan nail and FNS can transfer the stress on the femoral head to the femoral shaft, while fixation with 3 cannulated screws relies on the “three-point support principle”, which transfers torque through the interaction between the screw and the cancellous bone. InterTan nail is an intramedullary fixation device with main nail located in medullary cavity of the femoral shaft, and a short level arm, which can produce high mechanical strength upon A-P bending and axial loading. FNS is an extramedullary angular stabilization device. Although FNS can transfer stress to the femoral shaft, its force arm is long, this is the reason why FNS was inferior to InterTan nail in terms of axial stiffness and A-P bending stiffness. Additionally, considering the design of FNS (a short plate, bolt, locking screw, and anti-rotation screw), FNS is relative unstable (short bone plate) and weaker in the initial stability (fixed by few screw) than the InterTan nail. Our findings demonstrated superior fixation strength of FNS and InterTan nail compared with three cannulated screws. Under normal conditions, 1400 N–1500 N force was loaded to the hip joint during daily activities [17]. And instantaneous force loaded to the hip joint can also reach 1, 400 N during the postoperative recovery period involving full height bearing.

The overall deformation of the models and local deformation of implant holes can reflect displacement during the fracture healing process. In this study, correlation analysis revealed negative correlation between overall, local deformations and axial stiffness (Table 2). InterTan nail showed the shortest displacement, followed by FNS, and then the cannulated screws. The reason many be that InterTan nail can transfer the stress on the femoral head to the femoral shaft and its level arm is short. Meanwhile, InterTan nail may also have a tendency for a higher stiffness in treating patients with FNFs [16]. Using implants that have excellent biomechanical properties for internal fixation can limit the displacement of the fracture and better maintain the initial reduction.

In this study, we found an interesting phenomenon, i.e., the torsional stiffness of the FNS and InterTan nail was superior to that of cannulated screws, while there was no significant difference in the torsional stiffness between FNS and InterTan nail groups. Three cannulated screws are placed in parallel and distributed scatteredly close to the bone cortex, which can form a triangular support base with the largest area on lateral femoral wall, therefore, providing better holding power in the femoral head [18]. During rotation of the femoral head, the torque is eventually transferred to the lateral femoral cortex through the cannulated screws, thus producing anti-rotation effect. However, the ability of cannulated screws to resist torsion relying solely on the contact between the screw and cortical bone is poor [19]. In contrast, screws of FNS and InterTan nail within the femoral neck are connected with the bone plate and intramedullary nail, and finally fixed on the femoral shaft. The torque at the center of rotation of the femoral head is counteracted by the locking plate or intramedullary nail [20, 21]. Therefore, FNS and InterTan nail can both produce higher torsional stiffness than cannulated screws. Furthermore, the mechanical strength of internal fixation is related not only to the number of implants fixed on the bone, but also to the geometry of the fixation [19]. FNS and InterTan nail both have two screws located in the femoral head, their differences are that InterTan nail securely anchors the femoral head with a thick lag screw. The fully threaded compression screw underneath provides immediate compression of the fracture ends, and work together with lag screw to exert anti-rotation effect. However, the two screws in the femoral head are located close to each other with their tips approximating as a single point. In contrast, the bolt and threaded anti-rotation screw of FNS are cross-distributed at an angle of 7.5°, with their tips distributed scatteredly within the femoral head, thus increasing the holding power in the femoral head. Although the bolt of FNS is smooth and unthreaded, the ability of FNS to resist torsion is equivalent to that of InterTan nail. FNFs can only be healed by primary bone healing because the femoral neck lacks a periosteal covering [5], and proper micromotion contributes to fracture healing [9]. FNS allows interfragmentary micromotion, thus promoting fracture healing.

A biomechanical comparison of FNS and sliding hip screws, cannulated screws in unstable Pauwels III FNFs conducted by Stoffel et al. [14] showed that the biomechanical properties were comparable between FNS and sliding hip screws, FNS showed higher overall stability than cannulated screws. Rupprecht et al. [16] documented that InterTan nail provided increased axial compression stability compared to DHS and cannulated screws. Baitner et al. [22] and Samsami et al. [23] reported that the mechanical stiffness of DHS was superior to three inverted triangle screws. Aminian et al. [24] conducted a biomechanical analysis of four different fixation techniques in vertically oriented femoral neck, and found that DHS had stronger biomechanical stability than three cannulated screws. A systematic review documented that fixation with the DHS, locking plate, and proximal femoral nail were more effective than multiple screw fixation in Pauwels type III FNFs, and InterTan nail was more powerful than most other fixtures [25]. The biomechanical properties of different internal fixation methods and their advantages and disadvantage reported in the above-mentioned studies are similar to the findings of the present study. In addition, because both femoral retroversion and rotation are detrimental to fracture healing [8, 9], in this study, we also added the tests of the ability of the devices to resist A-P bending and rotation. To our knowledge, the current investigation of FNS is the first to demonstrate increased multidirectional stability without adverse effects by a modern treatment concept in multiple comparisons, identifying global biomechanical effects.

It is worth noting that the results from in vitro biomechanics studies should not be fully equivalent to the results of clinical treatment. However, the evidence from biomechanical studies plays an important role in the short-term safety assessment of implants for use in clinic [26]. Although InterTan nail had high axial compression stiffness, it does not have a dynamic sliding pressure function. From a biomechanical point of view, devices allowing dynamic compression may be less likely to cause fatigue failure of the implants and implant perforation [27, 28]. FNS can not only provide angular stability and rotational stability, but also has dynamic sliding pressure function. Its design is similar to DHS combined with anti-rotation screw. Regarding the long-term safety of the fracture healing process, clinical studies suggest that increasing fixation stability is important for fracture healing [29, 30]. Zhou et al. [31] reported that the nonunion rate after FNS was lower than that of traditional cannulated screws for femoral neck fractures. However, due to the short time on the market of FNS, there are few clinical studies investigating the efficacy of FNS in the treatment of FNFs, so it is impossible to confirm its effectiveness in actual clinical application. More clinical studies are needed to confirm the potential advantages of FNS observed in this biomechanical study.

This study has several limitations. First, we selected artificial composite bone for biomechanical testing: in comparison with human cadaveric femurs, artificial composite femurs have the advantages of being easier to store, cheaper and easier to obtain, and artificial composite femurs are not biohazardous with minimal variation between specimens. In addition, there are no statistically significant differences between artificial composite bone and human cadaveric femurs with respect to various mechanical stability parameters [32]. However, artificial composite bone may not reflect the mechanical behavior of femurs in individuals with osteoporosis, and further biomechanical studies using human cadaveric femurs are needed to explore the influence of the different implants on load distribution [33]. Second, in this study, we created idealized Pauwels type III FNF models by using oscillating saw, but the jagged features of bone fragments found in clinic may not be simulated. Therefore, compared with the actual clinical situation, this idealized model reduces the interface friction and thus underestimates the stiffness values, which may lead to the misinterpretation of the implants as having biomechanical properties in clinical applications. Third, we did not consider the influence of the muscle attachment points around the hip joint. The femoral neck is usually maintained in a compressed state due to the muscle contraction forces applied to the femur during normal gait [34]. Therefore, the stiffness values may be underestimated in this study. Fourth, in this study, we only performed quasi-static and dynamic cyclic loading tests, and did not perform overload destructive test. In this study, we found that local deformation and overall deformation are highly correlated, with most deformation occurring at the fracture site, and the fixation hole may be enlarged during removal of the implants, which can affect the accuracy of the measurement. So there is a need to conduct 3D motion and strain analysis in future studies by using some expensive equipment [24]. Last but not least, we only compared the biomechanical properties of FNS with the conventional three cannulated screws and InterTan nail in unstable FNFs, but did not compare its biomechanical stability with other fixation implants, such as locking plates. Further studies are required to confirm the advantages and disadvantages of FNS in comparison with other internal fixation devices.

## Conclusions

For the unstable FNFs, both FNS and InterTan nail showed higher stability compared with 3 cannulated screws. In terms of axial stiffness and A-P bending stiffness, InterTan nail was superior to FNS and 3 cannulated screws, and FNS was superior to 3 cannulated screws. FNS also showed torsional stiffness comparable to that of the InterTan nail. FNS, as a minimally invasive implant, its biomechanical advantages may be beneficial for clinical application, which is expected to be a promising internal fixation implant for fixation of unstable FNFs. However, further clinical studies are needed to confirm our findings.

## Material and methods

### Models establishment

**Fig. 3:**
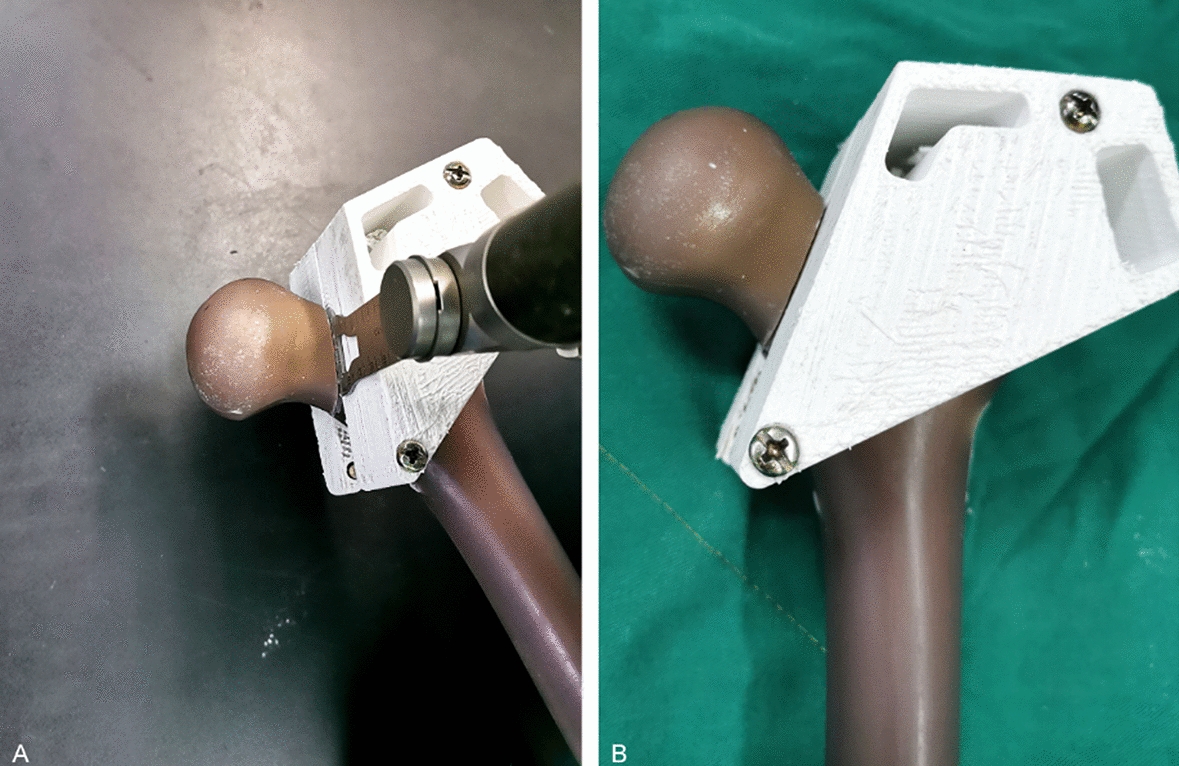
**A** Osteotomy was performed with an oscillating saw of 0.9 mm thickness under the guidance of the self-made 3D printed osteotomy mold. **B** Creating Pauwels type III femoral neck fracture models with an angle of 20° between the fracture line and the axis of the femoral shaft

A total of 18 left artificial femurs (model #3403, medium, Fourth Generation Composite Bone, Sawbones, Pacific Research Laboratories, Vashon, WA, USA) were selected and randomly divide into FNS group (group A, FNS, 130°, Depuy Synths, Switzerland), InterTan nail group (group B, InterTan nail, 125°, Smith & Nephew, USA), and cannulated screw group (group C, cannulated screws, 7.3 mm, Depuy Synths, Switzerland), with 6 specimens in each group. Specimen preparation and implant placement were performed by the same orthopedic surgeon to ensure consistency. Holes were predrilled in all specimens with a guide pin matching the implant prior to osteotomy in order to ensure standard placement of the implants under an anatomical reduction status after osteotomy. In groups A and B, guide pin was inserted along the central axis of the femoral neck in both the AP and later views by using a guide under G-arm fluoroscopy guidance, following the manufacturer’s instructions, then holes were predrilled. In group C, according to the position of cannulated screw placement recommended by Heetveld et al. [35] and Bosch et al. [36], three guide pins were inserted by a parallel guide at 125° with respect to the longitudinal axis of the femoral shaft in an inverted triangle configuration. After predrilling was finished (Fig. 3), osteotomy was performed at the middle of the femoral neck with an oscillating saw of 0.9 mm thickness under the guidance of the self-made 3D printed osteotomy mold. Pauwels type III FNF models with an angle of 20° between the fracture line and the axis of the femoral shaft were created [18]. The distal portions of all artificial femurs were excised at 10 cm from the femoral condyle.

### Fixation methods

**Fig. 4:**
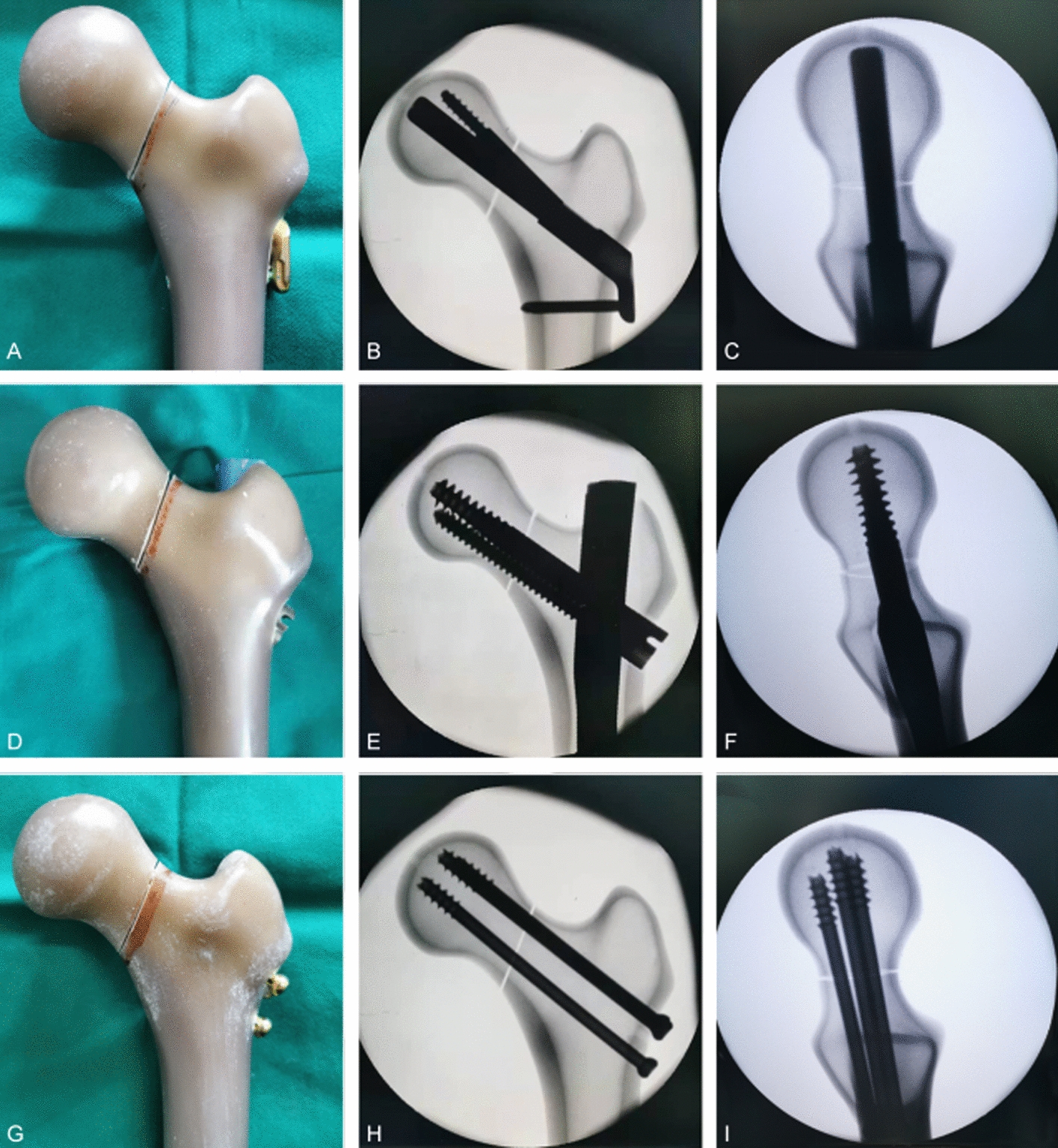
Fixation of Pauwels type III femoral neck fractures with three internal fixation implants. **A** Fixation with FNS, **B**, **C** anteroposterior and lateral fluoroscopic views after FNS fixation, **D** fixation with InterTan nail, **E**, **F** anteroposterior and lateral fluoroscopic views InterTan nail fixation, **G** fixation with three cannulated screws, **H**, **I** anteroposterior and lateral fluoroscopic views after fixation with three cannulated screws

To ensure standard placement of the implants within and between groups, implants were placed under G-arm fluoroscopy guidance. In group A, after enlarging holes under the guidance of the guide pin, special insertion instrument assembled with bolt and locking plate was inserted along the axis of the femoral neck until the tip reached to a distance of 5 mm distal from the subchondral bone, and the locking plate was placed on the longitudinal axis of the femoral shaft. The anti-rotation and locking screws were then inserted after enlarging the holes gradually under the guidance of a special guide, followed by applying pressure on the fracture ends. In group B, the guide pin of intramedullary InterTan nail was inserted slightly medial to the apex of the greater trochanter. The guide pin was at an angle of 4° to the femoral anatomical axis in the AP view, and was in line with the femoral medullary cavity in the lateral view. After reaming, the intramedullary nail was inserted, the lag screw guide pin was then inserted through the pre-drilled holes. After reaming, the lag screw was inserted along the axis of the femoral neck with a tip–apex distance less than 15 mm confirmed by fluoroscopy [37]. Through the guidance of the lag screw, compression screw was inserted to apply compression pressure on the fracture ends, followed by distal locking screw insertion. In group C, guide pins were inserted through the three pre-drilled holes to ensure anatomical reduction of the fracture. The fractures were fixed with 7.3 mm cannulated screw after enlarging the holes with a special cannulated drill bit. The three cannulated screws were inserted within 3 mm of the femoral neck cortex and parallel to each other within 10°, the tips of the screws were located within 5 mm of the subchondral bone of the femoral head [35]. In order to avoid the occurrence of “in–out–in”, the three screws were distributed in an inverted obtuse triangle configuration rather than an isosceles triangle [36] (Fig. 4).

### Biomechanical testing

The artificial femurs were mounted by a fixture attached to the base of an Instron E10000 testing machine (Instron Corporation, Norwood, MA, USA) with a measurement range of ± 10 KN, accuracy of ± 0.5%, torque measurement range of ± 100 Nm, and torque measurement accuracy of ± 0.5%. The loading and displacement were recorded by the software of the Instron E10000 testing machine.

### Quasi-static tests

**Fig. 5:**
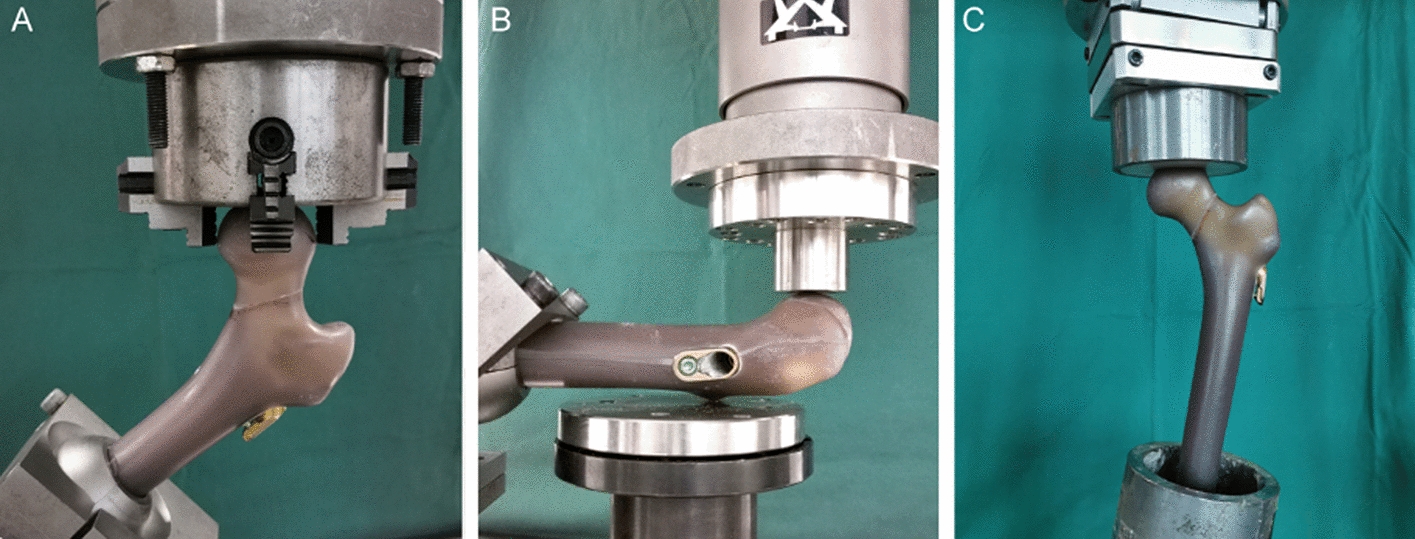
Biomechanical test setups. **A** Torsion test, the femurs were placed in an inclined fashion, torque around the central axis of the femoral neck was applied. **B** A-P bending test, the femur was placed horizontally, vertical compression force was applied to the front of the femoral head. **C** Axial compression test, femurs were positioned vertically in 10° adduction

According to the method reported by Zdero et al. [18], in order not to affect the subsequent tests, it was required to keep the artificial femur model undamaged during the tests and a non-destructive load level was chosen for quasi-static tests. During the torsion test, the femurs were placed in an inclined fashion: the distal end of each femur was fixed by a self-made fixture, the femoral head was fixed by a caliper, the femoral neck axis and rotation axis of the testing machine were in the same straight line (Fig. 5A). The test machine delivered torque at a torsional speed of 0.5°/s, the torque was then recorded until 10° rotation of the femoral head around the femoral neck axis reached. Counterclockwise rotation test was performed after the clockwise rotation test was completed. A study by Ragnarsson et al. [9] revealed that 10° rotation of the femoral head around the femoral neck axis can cause the occurrence of nonunion. During A-P bending test, the femur was placed horizontally, the distal end of the femur was fixed, and a support was placed below the lesser trochanter to let the femoral neck and head hang in the air [38], and the front of the femoral head was loaded to simulate forces applied to the hip joint during stand up from a chair [39] (Fig. 5B). During the axial compression test, the distal end of the femur was embedded in a mixture of epoxy resin (E-51, Shanghai, China) and hardener (593, beijing, China) and placed vertically in a special beveled fixture (the angle between the bevel of the fixture and the horizontal plane was 10°). The femurs were kept at 10° adduction to simulate the forces applied to the femur during single-leg stance [40]. The femoral head was loaded in compression along the machine axis via a spherically shaped polymethylmethacrylate shell cup attached to the machine actuator, and the low-friction shell cup avoided the accumulation of shear forces during compression (Fig. 5C). In both A-P bending and axial compression tests, a 30 N preload was applied, and the maximal load was set at 500 N with a loading rate of 3 mm/min, femoral retroversion and axial compression deformation were recorded. All static tests were repeated three times, the average value was then taken.

### Cyclic loading test

After the static test was completed, the femur was still positioned in the vertical orientation and subjected to axial dynamic cyclic test under sinusoidal load control. To prevent slipping of the femurs, the valley load was kept at a constant level of 60 N throughout the test. Subsequently, the dynamic load was applied at the femoral head with sinusoidal motion using load control (rate 2 Hz; cycles 10,000; maximum load 1400 N; preload 60 N) [41]. The 1400 N represents the measured force on the hip in a 70 kg person standing on one leg [42], and the number of cycles approximates the number of steps taken over a 4-week to 6-week period, i.e., the period of fracture healing [22, 43–45].

### Observation indicators

The average slope of the linear load–deformation curve obtained from quasi-static tests defines the initial torsional stiffness, A-P bending stiffness, and axial compression stiffness [18]. After the cyclic test was completed, the overall deformation of the models was estimated as the displacement recorded by the software of the mechanical testing apparatus. The overall deformation reflected the weight-bearing deformation during the fracture healing period. Local deformation was defined as interfragmental displacement. In order to assess local deformation, the maximum diameter of implant holes at the femoral head in the distal-to-proximal direction was measure by a Vernier caliper after the internal fixation implants were removed, then the mean value between the maximum diameter of the implants in the distal–proximal direction at the plane of osteotomy and the measured maximum diameter of the hole in the distal–proximal direction for each group was calculated [46]. In group C, the difference between the deformation of 3 implant holes was measured, the average value was taken, and the formation of the fracture line was considered as fixation failure.

### Statistical analysis

Statistical analysis was carried out using the SPSS software (version 24.0, IBM, New York, NY, USA). Data were tested for normality using the Kolmogorov–Smirnov test. After confirming data normality, the differences in axial stiffness, A-P bending stiffness, torsional stiffness, overall deformation, and local deformation of holes between 3 groups were analyzed using one-way analysis of variance (ANOVA) with Bonferroni post hoc tests for multiple comparisons. The 95% confidence interval was used for considering statistical significance. The level of significance was set at *P* < 0.05. Pearson’s correlation analysis was used to determine the correlation between axial stiffness, A-P bending stiffness, torsional stiffness, as well as between overall, local deformations and the axial stiffness after cyclic loading tests.

## Data Availability

All data generated or analyzed during this study are included in this published article.

## Abbreviations

FNFs: Femoral neck fractures
FNS: Femoral neck system
A-P: Anterior–posterior
ANOVA: One-way analysis of variance
DHS: Dynamic hip screw

## References

[1] Thorngren K, Hommel A, Norrman PO (2002). Epidemiology of femoral neck fractures. Injury.

[2] Parker MJ (2000). The management of intracapsular fractures of the proximal femur. J Bone Joint Surg Br.

[3] Slobogean G, Sprague SA, Scott T (2015). Complications following young femoral neck fractures. Injury.

[4] Giannoudis PV, Jones E, Einhorn TA (2011). Fracture healing and bone repair. Injury.

[5] Roerdink WH, Aalsma AM, Nijenbanning G (2009). The dynamic locking blade plate, a new implant for intracapsular hip fractures: biomechanical comparison with the sliding hip screw and twin hook. Injury.

[6] Zhang LL, Zhang Y, Ma X (2017). Multiple cannulated screws vs. dynamic hip screws for femoral neck fractures: a meta-analysis. Orthopade.

[7] Palm H, Gosvig K, Krasheninnikoff M (2009). A new measurement for posterior tilt predicts reoperation in undisplaced femoral neck fractures: 113 consecutive patients treated by internal fixation and followed for 1 year. Acta Orthop.

[8] Ragnarsson JI, Kärrholm J (1991). Stability of femoral neck fracture. Roentgen stereophotogrammetry of 29 hook-pinned fractures. Acta Orthop Scand.

[9] Ragnarsson JI, Kärrholm J (1992). Factors influencing postoperative movement in displaced femoral neck fractures: evaluation by conventional radiography and stereoradiography. J Orthop Trauma.

[10] Knobe M, Altgassen S, Maier KJ (2018). Screw-blade fixation systems in Pauwels three femoral neck fractures: a biomechanical evaluation. Int Orthop.

[11] Knobe M, Bettag S, Kammerlander C (2019). Is bone-cement augmentation of screw-anchor fixation systems superior in unstable femoral neck fractures? A biomechanical cadaveric study. Injury.

[12] Luttrell K, Beltran M, Collinge CA (2014). Preoperative decision making in the treatment of high-angle “vertical” femoral neck fractures in young adult patients. An expert opinion survey of the Orthopaedic Trauma Association’s (OTA) membership. J Orthop Trauma.

[13] Slobogean G, Stockton DJ, Zeng B (2017). Femoral neck fractures in adults treated with internal fixation: a prospective multicenter chinese cohort. J Am Acad Orthop Surg.

[14] Stoffel K, Zderic I, Gras F (2017). Biomechanical evaluation of the femoral neck system in unstable pauwels III femoral neck fractures: a comparison with the dynamic hip screw and cannulated screws. J Orthop Trauma.

[15] Ruecker AH, Rupprecht M, Gruber M, Gebauer M, Barvencik F, Briem D, Rueger JM (2009). The treatment of intertrochanteric fractures: results using an intramedullary nail with integrated cephalocervical screws and linear compression. J Orthop Trauma.

[16] Rupprecht M, Grossterlinden L, Ruecker AH (2011). A comparative biomechanical analysis of fixation devices for unstable femoral neck fractures: the Intertan versus cannulated screws or a dynamic hip screw. J Trauma.

[17] Duda GN, Schneider E, Chao EY (1997). Internal forces and moments in the femur during walking. J Biomech.

[18] Zdero R, Keast-Butler O, Schemitsch EH (2010). A biomechanical comparison of two triple-screw methods for femoral neck fracture fixation in a synthetic bone model. J Trauma.

[19] Brattgjerd JE, Loferer M, Niratisairak S (2018). Increased torsional stability by a novel femoral neck locking plate. The role of plate design and pin configuration in a synthetic bone block model. Clin Biomech (Bristol, Avon).

[20] Basso T, Klaksvik J, Foss OA (2014). The effect of interlocking parallel screws in subcapital femoral-neck fracture fixation: a cadaver study. Clin Biomech (Bristol, Avon).

[21] Basso T, Klaksvik J, Foss OA (2014). Locking plates and their effects on healing conditions and stress distribution: a femoral neck fracture study in cadavers. Clin Biomech (Bristol, Avon).

[22] Baitner AC, Maurer SG, Hickey DG (1999). Vertical shear fractures of the femoral neck. A biomechanical study. Clin Orthop Relat Res.

[23] Samsami S, Saberi S, Sadighi S (2015). Comparison of three fixation methods for femoral neck fracture in young adults: experimental and numerical investigations. J Med Biol Eng.

[24] Aminian A, Gao F, Fedoriw WW (2007). Vertically oriented femoral neck fractures: mechanical analysis of four fixation techniques. J Orthop Trauma.

[25] Cha YH, Yoo JI, Hwang SY (2019). Biomechanical evaluation of internal fixation of pauwels type III femoral neck fractures: a systematic review of various fixation methods. Clin Orthop Surg.

[26] Schemitsch EH, Bhandari M, Boden SD (2010). The evidence-based approach in bringing new orthopaedic devices to market. J Bone Joint Surg Am.

[27] Berkes M, Little MT, Lazaro LE (2012). Catastrophic failure after open reduction internal fixation of femoral neck fractures with a novel locking plate implant. J Orthop Trauma.

[28] Biber R, Brem M, Bail HJ (2014). Targon Femoral Neck for femoral-neck fracture fixation: lessons learnt from a series of one hundred and thirty five consecutive cases. Int Orthop.

[29] Alshameeri Z, Elbashir M, Parker MJ (2017). The outcome of intracapsular hip fracture fixation using the Targon Femoral Neck (TFN) locking plate system or cannulated cancellous screws: a comparative study involving 2004 patients. Injury.

[30] Yin H, Pan Z, Jiang H (2018). Is dynamic locking plate(Targon FN) a better choice for treating of intracapsular hip fracture?. A meta-analysis Int J Surg.

[31] Zhou XQ, Li ZQ, Xu RJ (2021). Comparison of early clinical results for femoral neck system and cannulated screws in the treatment of unstable femoral neck fractures. Orthop Surg.

[32] Papini M, Zdero R, Schemitsch EH (2007). The biomechanics of human femurs in axial and torsional loading: comparison of finite element analysis, human cadaveric femurs, and synthetic femurs. J Biomech Eng.

[33] Basso T, Klaksvik J, Syversen U (2014). A biomechanical comparison of composite femurs and cadaver femurs used in experiments on operated hip fractures. J Biomech.

[34] Duda GN, Heller M, Albinger J (1998). Influence of muscle forces on femoral strain distribution. J Biomech.

[35] Heetveld M, Raaymakers EL, Luitse JS (2007). Rating of internal fixation and clinical outcome in displaced femoral neck fractures: a prospective multicenter study. Clin Orthop Relat Res.

[36] Bosch U, Schreiber T, Krettek C (2002). Reduction and fixation of displaced intracapsular fractures of the proximal femur. Clin Orthop Relat Res.

[37] Baumgaertner M, Curtin SL, Lindskog DM (1995). The value of the tip-apex distance in predicting failure of fixation of peritrochanteric fractures of the hip. J Bone Joint Surg Am.

[38] Kauffman JI, Simon JA, Kummer FJ (1999). Internal fixation of femoral neck fractures with posterior comminution: a biomechanical study. J Orthop Trauma.

[39] Yoshioka S, Nagano A, Himeno R (2007). Computation of the kinematics and the minimum peak joint moments of sit-to-stand movements. Biomed Eng Online.

[40] Bergmann G, Deuretzbacher G, Heller M (2001). Hip contact forces and gait patterns from routine activities. J Biomech.

[41] Gueorguiev B, Ockert B, Schwieger K (2011). Angular stability potentially permits fewer locking screws compared with conventional locking in intramedullary nailed distal tibia fractures: a biomechanical study. J Orthop Trauma.

[42] Denham RA (1959). Hip mechanics. J Bone Joint Surg Br.

[43] Kubiak EN, Bong M, Park SS (2004). Intramedullary fixation of unstable intertrochanteric hip fractures: one or two lag screws. J Orthop Trauma.

[44] Laursen JO (1999). Treatment of intracapsular fractures of the femoral neck in Denmark: trends in indications over the past decade. Acta Orthop Belg.

[45] Springer ER, Lachiewicz PF, Gilbert JA (1991). Internal fixation of femoral neck fractures. A comparative biomechanical study of knowles pins and 6.5 mm cancellous screws. Clin Orthop Relat Res.

[46] Kuan F, Hsu KL, Lin CL (2019). Biomechanical properties of off-axis screw in pauwels III femoral neck fracture fixation: bicortical screw construct is superior to unicortical screw construct. Injury.

